# MDCFVit-YOLO: A model for nighttime infrared small target vehicle and pedestrian detection

**DOI:** 10.1371/journal.pone.0324700

**Published:** 2025-06-17

**Authors:** Huiying Zhang, Qinghua Zhang, Yifei Gong, Feifan Yao, Pan Xiao

**Affiliations:** College of Information and Control Engineering, Jilin Institute of Chemical Technology, Jilin, China; Hohai University, CHINA

## Abstract

An MDCFVit-YOLO model based on the YOLOv8 algorithm is proposed to address issues in nighttime infrared object detection such as low visibility, high interference, and low precision in detecting small objects. The backbone network uses the lightweight Repvit model, improving detection performance and reducing model weight through transfer learning. The proposed MPA module integrates multi-scale contextual information, capturing complex dependencies between spatial and channel dimensions, thereby enhancing the representation capability of the neural network. The CSM module dynamically adjusts the weights of feature maps, enhancing the model of sensitivity to small targets. The dynamic automated detection head DAIH improves the accuracy of infrared target detection by dynamically adjusting regression feature maps. Additionally, three innovative loss functions—focalerDIoU, focalerGIOU and focalerShapeIoU are proposed to reduce losses during the training process. Experimental results show that the detection accuracy of 78% for small infrared nighttime targets, with a recall rate of 58.6%, an mAP value of 67%. and a parameter count of 20.9M for the MDCFVit-YOLO model. Compared to the baseline model YOLOv8, the mAP increased by 6.4%, with accuracy and recall rates improved by 4.5% and 5.7%, respectively. This research provides new ideas and methods for infrared target detection, enhancing the detection accuracy and real-time performance.

## 2. Introduction

With the rapid advancement of technology, fields such as autonomous driving, intelligent transportation systems, and security monitoring are placing increasing demands on the accuracy and real-time performance of target detection. Infrared target detection, as a crucial technological tool, has unique advantages in nighttime and low-light environments, effectively overcoming the limitations of visible light imaging in such conditions. Consequently, infrared target vehicle and pedestrian detection have become a key focus of current research. In the field of target detection, the YOLO(You Only Look Once) series of algorithms is highly regarded for its efficiency and accuracy. As the member of the YOLO series, YOLOv8 inherits the advantages of its predecessors and introduces innovations in the backbone network, detection head, and loss functions, greatly enhancing the flexibility and performance model. This makes YOLOv8 a powerful tool for achieving high-precision infrared target detection. However, despite the significant accomplishments of YOLOv8 in target detection, challenges remain in the realm of infrared target detection. Infrared images often have lower contrast and higher noise levels, making it challenging for traditional target detection algorithms to effectively extract and identify target features. Additionally, the lack of clear distinction between vehicle and pedestrian targets and the background in infrared images further complicates detection. To address these challenges, this paper proposes a method for infrared target vehicle and pedestrian detection based on the YOLOv8 model. This method enhances YOLOv8 of the backbone network and detection head to better suit the characteristics of infrared images, improving the extraction and recognition of target features. Additionally, a self-developed model is introduced to reduce noise interference and enhance the contextual relationship of images, thereby further improving detection performance.

Nighttime environments pose a great challenge to target detection due to the lack of visible light, and infrared technology has become an important means of solving this problem by virtue of its all-weather operation. Infrared target detection can capture target information under no light or low light conditions by sensing the infrared radiation emitted by the object, which is widely used in the fields of security, automatic driving, rescue and so on. Especially in night scenes, infrared target detection not only makes up for the shortcomings of visible light systems, but also attracts attention for its ability to penetrate haze and soot. In this paper, we will systematically discuss the research and application of infrared target detection at night from the aspects of technical background, current status, challenges and future development direction.

The basis of infrared target detection lies in the imaging principle of infrared radiation. Infrared sensors achieve target identification by detecting the radiation difference between the target and the background and converting it into a grey scale image. While traditional visible light cameras are difficult to work in nighttime environments due to the lack of natural light, infrared imaging technology is not subject to this limitation. According to the wavelength range, infrared technology is mainly classified into near infrared (0.75–1.4 μm), mid-infrared (3–5 μm), and long-wave infrared (8–14 μm), of which long-wave infrared is the most widely used in nighttime detection due to its high sensitivity to thermal radiation. Traditional infrared target detection relies on signal processing methods, such as threshold-based segmentation and filter enhancement. However, these methods have limited effect in complex background or low signal-to-noise ratio conditions. With the development of artificial intelligence, especially deep learning technology, infrared target detection has entered a new stage. Neural networks can automatically extract target features and significantly improve detection accuracy and robustness, which has become a hot spot in current research.

In recent years, infrared target detection at night has made great progress, and infrared target detection algorithms based on models such as convolutional neural network (CNN) and Transformer have significantly improved detection performance.Frameworks such as YOLO and Faster R-CNN are widely adapted to infrared image processing, and a series of improvement schemes are proposed for the detection of weak targets at night, such as multi-scale feature fusion and attention mechanism. A single infrared information may not be sufficient to meet all the challenges in a night scene, so fusion with multi-sensor data, such as visible light and radar, has become a trend. The fusion of infrared and visible light images utilises the thermal imaging properties of infrared and the detailed information of visible light to further improve the accuracy of target detection.

With the enhancement of hardware arithmetic power, such as the popularity of GPUs and TPUs, the lightweight network design MobileNet makes real-time infrared target detection possible. Some algorithms are still able to achieve processing speeds of more than a hundred frames per second in complex scenarios, meeting the needs of night surveillance and autonomous driving. For the construction of datasets, the lack of infrared target detection datasets has been the bottleneck, but in recent years several public datasets (FLIR, NUDT-SIRST) have been gradually released, which provides support for algorithm training and validation. Nighttime infrared target detection in the application field, in security, city security, border protection and other fields are widely used infrared cameras to achieve 7 × 24 hour all-weather monitoring. Infrared technology is able to penetrate darkness and haze and provide clear target images. In the field of autonomous driving, at night and in bad weather, infrared thermal imaging technology provides reliable sensing capabilities for autonomous vehicles, helping to identify potential obstacles such as pedestrians and vehicles. In emergency rescue, infrared technology can detect the location of trapped people in fire, earthquake and other scenarios to assist rescue operations.

Currently, infrared target detection at night mainly focuses on the detection of weak targets The targets in infrared images at night are often small in size, weak in brightness, and lack texture and shape information. Researchers have improved the detection capability through multi-scale feature extraction and attention mechanism. By suppressing the complex background, night scenes may contain background noise such as clouds and heat source interference, so how to effectively separate the target from the background is the focus of the research. By optimising the low signal-to-noise ratio, infrared images have low resolution and poor contrast, to address this problem, data enhancement and loss function optimisation (e.g., Focal Loss) have become the direction of improvement.

However, the detection of infrared targets at night is also facing many problems. Infrared images lack colour and detail information, and the targets usually appear as fuzzy hot spots, which makes it difficult to distinguish between target types, especially in long-distance scenes at night. In the nighttime environment, ground heat reflection, natural heat sources and even atmospheric scattering can introduce noise and increase the false alarm rate. Compared to the visible light domain, the infrared dataset has a small sample size, a single scene, and the annotation accuracy needs to be improved. Although the deep learning model has excellent performance, it is computationally intensive, and its deployment on embedded devices still needs to be optimised to meet real-time requirements. Weather changes at night can have an impact on infrared radiation, leading to fluctuations in detection performance.

In response to these challenges, the main contributions of this paper include, firstly, a detailed analysis of the challenges and difficulties of infrared target detection and an exploration of the strengths and limitations of the YOLOv8 model in this context; secondly, through the introduction of a new lightweight backbone network Repvit, Dynamic Automated Inspection Head (DAIH), self-developed models of context-sensitive modulation (CSM) and Multiplexed Interactive Attention (MIA) modules, the MDCFVit-YOLO model based on the YOLOv8 architecture is proposed, and in order to improve the detection effect, a new loss function is also used in this paper. focalerShapeIoU, the MDCFVit-YOLO model in this paper significantly improves the accuracy and real-time performance of infrared target vehicles and pedestrians detection; finally, we experimentally validate the effectiveness of the proposed method and compare it with other advanced infrared target detection algorithms. The experimental results show that MDCFVit-YOLO achieves 78% small target detection accuracy, 58.6% recall, and 67% mAP on the FLIR_ADAS_v2 dataset, which is an improvement of 4.5%, 5.7%, and 6.4%, respectively, compared to the baseline model YOLOv8. The number of parameters is 20.9M and the frame rate is 65.2 FPS, indicating that the model maintains real-time performance while improving performance. These specific metrics fully validate the claimed performance improvements and provide new ideas and methods for infrared target detection.

Compared with the existing methods, the MDCFVit-YOLO model proposed in this paper demonstrates novelty in the following aspects: the MPA module breaks through the limitation of the traditional attention mechanism that is difficult to capture the interactions between spatial and channel dimensions at the same time; the CSM module enhances the feature expression capability through multi-level contextual aggregation; and the combination of DAIH detector head and dynamic tuning strategy improves the detection performance of the complex scenarios significantly. detection performance in complex scenarios; the new loss function is proposed, and the focalerShapeIoU breaks through the inadequacy of the traditional IoU loss for small target localisation, and together these improvements provide a solution to the problems of low visibility, high interference and low detection accuracy of small targets in IR detection. This research will provide new insights and methods in the field of infrared target vehicle and pedestrian detection, contributing to the technological advancement and social development in related fields.

## 3. Related work

Nighttime driving infrared image target detection is a crucial topic, aiming to accurately identify and locate vehicles and pedestrians in images using advanced image processing techniques.

Deep learning technology has become dominant in the field of infrared vehicle and pedestrian detection. This technology constructs complex neural network models and uses back-propagation algorithms to optimize network parameters, allowing computers to automatically learn and extract object features from images. Currently, deep learning-based object detection algorithms can be broadly divided into two categories: two-stage detection algorithms and single-stage detection algorithms.

Two-stage detection algorithms, such as the R-CNN series (including R-CNN [1], Fast R-CNN [2], and Faster R-CNN [3]) are renowned for their high accuracy. These algorithms first extract image features through neural networks, then generate candidate regions, and classify and localize these regions. Although they excel in detection accuracy, they are generally slower in processing speed, which may make them unsuitable for real-time detection scenarios.

To address the speed deficiencies of two-stage detection algorithms, researchers have proposed single-stage detection algorithms, such as the YOLO series (including YOLOv1 [4], YOLOv2 [5], etc.) and the SSD series [6]. These techniques use neural networks to directly extract picture information and simultaneously carry out object categorization and localization, significantly enhancing detection speed. While single-stage detection algorithms may be slightly less accurate than two-stage algorithms, they excel in real-time detection tasks.

In infrared target detection, the attention mechanism enables the model to focus on key parts of the input information, thus significantly improving the model’s ability to handle complex tasks. Zhang [7] proposed an attention-guided pyramid context network (AGPCNet), which enhances the accuracy of small target infrared detection through the context pyramid module and the asymmetric fusion module, and the experimental results show that AGPCNet achieves an mIoU of 0.7288 and an F1 score of 0.8431 on the SIRST Aug dataset, and on the MDFA dataset The mIoU reached 0.4843 and the F1 score was 0.6525, with AUCs as high as 0.9344 and 0.8682, showing excellent detection of small targets in complex backgrounds. Su [8] designed an Attention Fusion Feature Pyramid Network (AFFPN) by introducing an expansive spatial pyramid pooling module in the feature extraction phase and enhancing the infrared target feature representation through the attention fusion module, which achieved an F1 score of 0.892 and an AUC of 0.956 on the SIRST dataset, and an F1 score of 0.788 on the MDFA dataset and an AUC of 0.901, proving its superiority in feature enhancement.

In infrared vehicle and pedestrian detection, small targets face challenges such as scarce feature information, complex and variable backgrounds, and unpredictable motion trajectories, which significantly increase the detection difficulty. However, scholars have continuously invested in research to explore new methods and theories to break through the bottleneck of small target detection and improve the detection accuracy and robustness. Ming [9] proposed an attention-based context-aware network (ACANet), which utilises the attention mechanism to extract features from local and global contexts to improve the performance of infrared small target detection, and it outperforms the traditional methods in terms of detection probability and mIoU metrics, demonstrating the strong potential of context-awareness. Zuo [10] designed an Attention Fusion Feature Pyramid Network (AFFPN), which significantly improves the performance of small target infrared detection by expanding the spatial pyramid pooling and attention fusion module, and the results are consistent with Su [8], with an F1 score of 0.892 and an AUC of 0.956 on the SIRST dataset, which verifies the robustness of the method. Ling [11] proposed an attention-based context-aware network to improve the accuracy of infrared small target detection by extracting features from local and global contexts, and although specific data are not available at the moment, its design idea is similar to that of Ming [9], and it is expected to perform well. Thin [12] proposed a Dense Nested Attention Network (DNAN), which significantly enhances IR small target detection through hierarchical attention mechanism and feature fusion strategy, and outperforms traditional methods on datasets such as NUDT-SIRST, with specific metrics such as mIoU and F1 scores showing the efficiency of hierarchical feature fusion.

In infrared vehicle and pedestrian detection, the design of the loss function is crucial to improve the detection performance. Conventional loss functions may perform poorly when dealing with small target infrared detection, so researchers have proposed improvements to optimise target detection and significantly improve overall detection performance. Among the loss function designs, the focal loss function (Focal) loss) significantly improves the detection performance of difficult-to-classify targets by reducing the weights of easy-to-classify samples [13]. Experiments show that the focal loss is excellent in dealing with class imbalance problems, especially in infrared small target detection, which can effectively improve the model’s focus on small targets. Yu [14] proposed a method combining multi-dimensional feature fusion and local detection enhancement for small target infrared detection, and improved the loss function to enhance the detection performance. Although specific values are not available, the method is expected to provide significant improvement in detection accuracy by optimising feature extraction and loss calculation. Another method to enhance the performance of IR target detection is to use an alternative model by replacing the pixel-level discrimination with a masked post-detection distribution model and employing a diffusion model to enhance the accuracy of small-target IR detection [15]. It has been shown that the diffusion model significantly improves the detection performance on the public dataset through data enhancement, e.g., the leakage and false detection rates are reduced in complex backgrounds.

In infrared target detection process, the modification of the backbone part and optimisation of the loss function are also crucial to improve the detection performance. Wang [16] proposed a modified backbone feature extraction network and repaired the original GIoU loss using Inner-GIoU loss, thus improving the speed and accuracy of small target detection. Experiments show that Inner-GIoU, through the auxiliary bounding box bounding box regression, ends up in accelerating the GIoU loss in small target detection by about 10% of the speed of convergence, and detection is also improved.

In the field of infrared target detection, improving the YOLO algorithm to enhance the detection performance is a commonly used and efficient method.YOLO-ISTD: A YOLOv5-S based significantly improves the detection performance of infrared small targets by replacing the original 8x, 16x, and 32x downsampling detector heads with 4x, 8x, and 16x downsampling detector heads. On the IRSTD-1K dataset, the method resulted in a 2.3% improvement in mAP and a 2.7% improvement in F1 score, which helped the model to better capture small target features and thus improve detection accuracy. Another study proposed a YOLOv5 infrared small target detection algorithm (YOLO-FR) based on a feature reorganisation sampling method [17]. By adjusting the feature map size without increasing or decreasing the amount of current feature information, the algorithm proposes the STD module and CARAFE operation to optimise the detection head structure, which achieves 97.4% of mAP50 in experiments, which is 7.4% higher than the original network, and further improves the detection accuracy. The method can effectively use the feature information in infrared images to significantly enhance the model’s detection ability for small targets.

The YOLOv7-based infrared small target network (YOLO-SDLUWD) better adapts the model to infrared image characteristics and improves the small target detection performance through the optimised detection head and feature fusion method [18]. On the public dataset, the method achieves a mAP of 85.3%, which is 2.7% higher than that of the original YOLOv7, and a frame rate of 68.9 YOLO and ConvNeXt-based Infrared Target Detection Networks (YOLO-CIR) improves the YOLO’s detector head structure and the feature implementation module to adapt to the high bit-width infrared images [19]. On the FLIR dataset, mAP50 is 3% higher than YOLOv5 and 5.6% higher than Faster R-CNN, and the number of parameters and computation are also significantly reduced, enabling the model to better capture the target features in the infrared images and thus improve its detection accuracy.

Aiming at the practical limitations of edge devices, a lightweight infrared target detection technique, Edge-YOLO, is proposed [20]. This method optimises the detection head of edge devices by replacing the backbone network of the YOLOv5m model and introducing a lightweight visual attention mechanism. AP50:95 reaches 50.6% on the COCO2017 dataset with an AP50 of 69.8% and 44.8% on VisDrone2019-DET, with a frame rate that meets real-time requirements (FPS ≥ 30). This approach reduces computational complexity and model size while maintaining high detection accuracy, providing an effective solution for IR target detection on edge devices.

To solve the safety problem of vehicles traveling at night in the field of automatic driving, an MDCFVit-YOLO algorithm is proposed to solve the problem of false detection and missed detection of infrared target images at night due to the low brightness and small target. The innovations are as follows:

1. To solve the problem that traditional attention mechanisms make it difficult to capture long-range dependencies and interactions between spatial and channel dimensions simultaneously, the MPA module is proposed in this paper, which generates more accurate attention graphs by integrating operations such as global average pooling, horizontal and vertical average pooling, therefore, the ability to capture feature graph details and characterize the network is improved.
2. To improve the network of ability to detect complex backgrounds and tiny targets, this paper proposes the Contextual Focus Modulation Mechanism (CSM), a mechanism that dynamically adjusts the weights of the feature maps so that the network pays more attention to important contextual information.
3. A new detection head DAIH is proposed. Through its unique task decomposition dynamic convolution strategy and unique architecture design, it can capture critical information in complex scenarios produce accurate detection results, and improve the adaptability and robustness of the regression features.
4. Innovatively proposed three kinds of loss functions: focalerGIOU combines IoU and minimum closed frame area to improve global positioning accuracy; focalerDIoU introduces centroid distance to significantly improve positioning accuracy; and focalerShapeIoU combines IoU, centroid distance, and shape matching to optimize the objective in multi-dimension.

## 4. Model architecture and methodology

### 4.1 YOLOv8 overview

**Fig 1:**
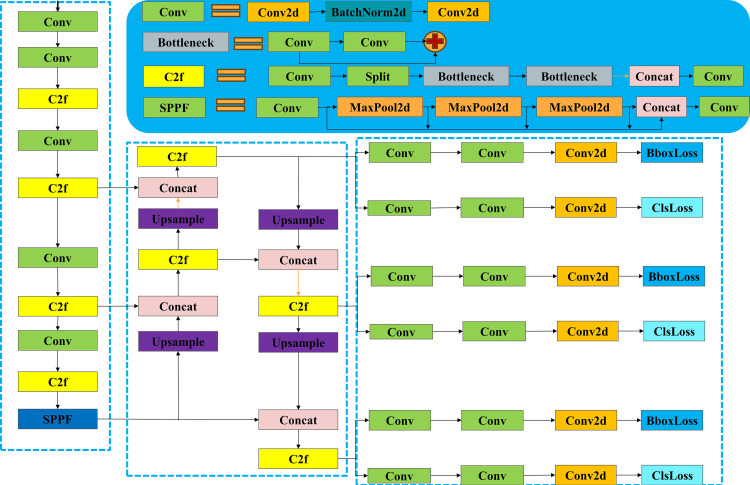
Block diagram of YOLOv8 model architecture.

The YOLOv8 algorithm introduces a new SOTA model, and YOLOv8 also offers models of different scales: N/S/M/L/X, to meet various application needs. The YOLOv8 architecture also consists of three parts: the backbone network, the neck network, and the head network. As shown in Fig 1, the backbone network is mainly composed of Conv modules, c2f modules, and SPPF modules. The neck network adopts a Feature Pyramid Network (FPN) structure [21] to achieve multi-scale object detection from top to bottom. The head network includes three object detectors, using grid-based anchors to perform detection on feature maps of different scales. As the feature map increases in size, it contains more contours and geometric information, which is beneficial for detecting small objects.

The Conv module is the basic component of many important modules. The C2f module includes Conv and Bottleneck modules. The SPPF module consists of Conv modules, Maxpool layers, and Concat layers.

### 4.2 MDCFVit-YOLO network architecture

The study adheres to all relevant ethical standards and publication guidelines. It has not been previously published or disseminated in any form, and all appropriate publication ethics have been strictly observed. Furthermore, the research complies with the requirements set forth by the Ethics Review Board or other relevant ethics committees, and no ethical or moral concerns are associated with the conduct of this study.

**Fig 2:**
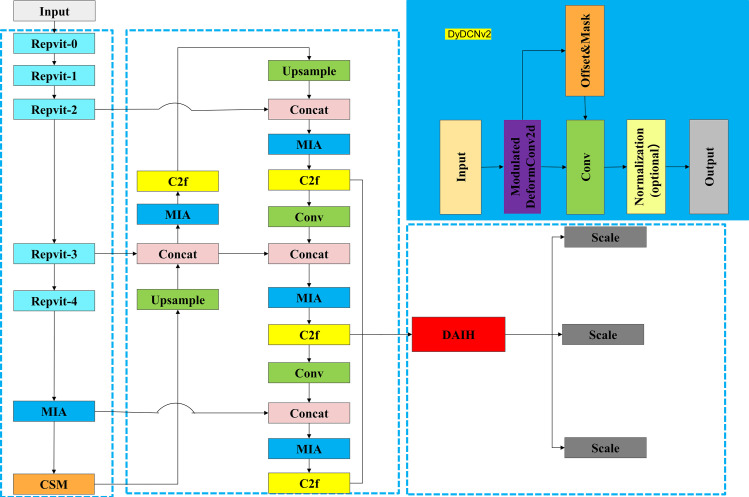
Block Diagram of MDCFVit-YOLO Network Architecture.

The backbone network of the MDCFVit-YOLO architecture uses Tsinghua University’s lightweight repvit network [22] as the Backbone to achieve network lightweight. The MDCFVit-YOLO network architecture is shown in Fig 2. Context-sensitive modulation (CSM) is also employed to enhance the network’s expressive capabilities. To integrate spatial and channel attention and enhance the representational capacity of the neural network, Multipath Interactive Attention (MIA) is used to simultaneously capture long-range dependencies and interactions between spatial and channel dimensions. For the detection head, Dynamic Automated Inspection Heads (DAIH) are used, enabling the detection head to provide efficient and accurate results in complex detection tasks. The DyDCNV2 dynamic convolution module used in the detection head makes regression features more adaptive and robust, as shown in the upper right part of the module in Fig 2.

The MDCFVit-YOLO model processes infrared images for high-precision night-time small target detection by taking the input images and normalising and optimising them through pre-processing techniques such as resizing, normalisation and data augmentation to enhance the robustness and generalisation capability of the model. The pre-processed images are then fed into a lightweight RepViT backbone network to extract multi-scale feature maps that capture key information. Then it is to enter these feature maps into a neck network such as Feature Pyramid Network (FPN) for multi-level feature fusion to enhance the detection of targets of different sizes. The fused feature maps are then passed through the multipath interactive attention (MPA) module to generate an attention map to highlight key regions using global pooling and horizontal and vertical average pooling operations, and then the weights are dynamically adjusted through the context-sensitive modulation (CSM) module to focus on important contextual information such as small targets and complex backgrounds. Afterwards, the enhanced feature maps are fed into the Dynamic Automated Inspection Head (DAIH) to generate classification and regression predictions via task decomposition, where the regression part uses dynamic convolution (DyDCNV2) to adjust the features and output the target bounding box, and the classification part generates the category probabilities. Finally, innovative loss functions (e.g. focalerDIoU) are used to optimise the parameters during training, and redundant frames are removed by non-maximal suppression (NMS) in the post-processing stage to filter out high-quality detection results. This process effectively overcomes the challenges of low visibility and high interference in nighttime infrared imagery through modular design and innovative technology to achieve efficient and accurate small target detection.

#### Research on multiplexed interactive attention.

**Fig 3:**
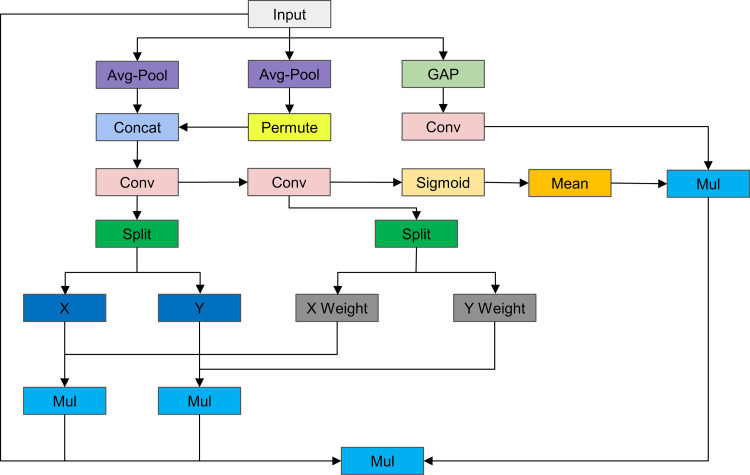
Architecture of the Multipath Interactive Attention Mechanism Model.

The proposed MIA (Multipath Interactive Attention-MIA) aims to enhance the representational capability of neural networks by integrating spatial and channel attention. Traditional attention mechanisms often struggle to simultaneously capture long-range dependencies and interactions between spatial and channel dimensions. MIA addresses this issue with an innovative approach, allowing the network to perform better in complex visual tasks. The architectural diagram of the MIA module is shown in Fig 3.

The MIA module mainly consists of global average pooling (GAP), horizontal and vertical average pooling, and a series of convolution operations to generate spatial and channel attention maps. The main function of the GAP path is to capture the global context information of the input feature map. Specifically, adaptive average pooling is applied to the input feature map, reducing it to one spatial point (1x1) per channel, then a convolution layer transforms the pooled features to enhance their representational ability for global dependencies. This step effectively extracts global features, providing critical information for subsequent feature fusion. The horizontal and vertical pooling paths capture spatial dependencies along the height and width dimensions, respectively. The input feature map is first subjected to adaptive average pooling along the height and width directions. To align the dimensions, the vertically pooled features need to undergo a dimension permutation. Then, the horizontally and vertically pooled features are concatenated along the channel dimension and processed through a convolution layer to generate a combined feature map. The combined feature map is then split back into separate horizontal and vertical components. This step aims to capture the spatial dependencies of the input feature map in different directions, thereby increasing the sensitivity of the attention mechanism to spatial features. To refine the spatial attention map, MIA further generates and processes attention weights. Specifically, another convolution layer is applied to the combined feature map, and the attention weights are generated through a sigmoid activation function. These weights are then split into horizontal and vertical weights. The original pooled features are element-wise multiplied with their corresponding weights, thereby modulating these features. Simultaneously, the global context features are modulated by the average value of the spatial weights. This process ensures the effective fusion of spatial and channel features, allowing the attention mechanism to more accurately capture long-range dependencies and global context information. The multi-path processed feature map is obtained by element-wise multiplying the input feature map with the sigmoid-activated horizontal and vertical features, and the globally modulated features, resulting in the final attention-modulated feature map. This process achieves an effective combination of spatial and channel attention, significantly enhancing feature representation ability.

The MIA mechanism effectively enhances the representational capability of neural networks by integrating multi-scale contextual information and capturing complex dependencies between spatial and channel dimensions. Through adaptive pooling and convolution operations, MIA generates precise attention maps, thus performing excellently in various visual tasks. This method provides a powerful solution for addressing the challenges faced by deep learning models in capturing long-range dependencies.

#### Proposed dynamic automated inspection head.

The existing object detection algorithms, such as YOLOv8, perform excellently on visible light images, but their detection performance on infrared images still needs improvement. Therefore, to better meet the needs of infrared object detection, this study modifies the detection head of YOLOv8 to enhance its performance in infrared images.

**Fig 4:**
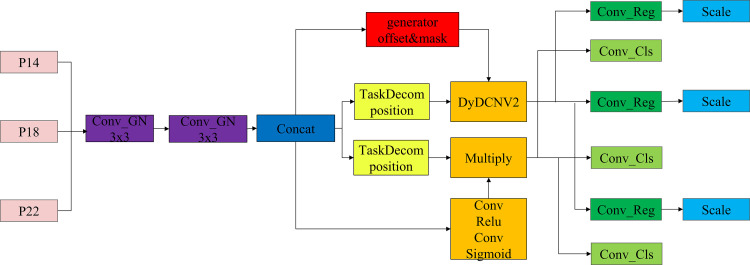
The architecture of dynamic automated Inspection head.

The architecture of the DAIH(dynamic automated Inspection head) module, as shown in Fig 4, primarily consists of feature extraction, task decomposition, dynamic convolution, feature alignment, and output modules. Multi-scale feature maps (P14, P18, P22) undergo feature extraction through a shared convolutional layer. This shared convolutional layer contains two convolutional layers (Conv_GN 3x3), which process the input feature maps separately. The purpose of this process is to reduce noise and extract more representative features. After feature extraction, the extracted feature maps are concatenated during the feature fusion phase to generate a composite feature map. The composite feature map is then decomposed by the task decomposition module into classification and regression feature maps. The task decomposition module (TaskDecomposition) adapts the feature maps based on the global average pooling results of the input feature map, generating feature maps suitable for classification and regression tasks.

Subsequently, the regression feature map is processed by the dynamic convolution module (DyDCNV2). DyDCNV2 dynamically adjusts the regression feature map using offsets and masks generated by a convolutional layer, enabling the feature map to better adapt to the regression task. Meanwhile, the classification feature map is processed through two convolutional layers to generate the classification probability map, which is then activated by the sigmoid function. The processed classification and regression feature maps are output through convolutional layers (Conv_Cl, Conv_Reg) as classification and regression results. These results are adaptively adjusted through the Scale module, ensuring consistency in feature maps across different resolutions.

Each module in the DAIH detection head has a clear function and collaborates to achieve efficient object detection. The Conv_GN 3x3 convolutional layers improve feature extraction and reduce noise in the feature maps through Group Normalization. The task decomposition module adaptively adjusts and decomposes the composite feature map into feature maps suitable for classification and regression tasks. The DyDCNV2 dynamic convolution module utilizes offsets and masks for dynamic adjustment of the feature map, enhancing the adaptability and robustness of the regression features. Finally, the Scale module adjusts the output feature maps, ensuring their consistency across different resolutions.

In the actual workflow, the input multi-scale feature maps (P14, P18, P22) first undergo feature extraction through the shared convolutional layer. After the feature fusion phase, a composite feature map is generated. This composite feature map is decomposed by the task decomposition module into classification and regression feature maps. The regression feature map generates offsets and masks through a convolutional layer and undergoes dynamic convolution with the DyDCNV2 module, producing the final regression feature map. Simultaneously, the classification feature map generates a classification probability map through two convolutional layers, followed by sigmoid activation. The classification and regression feature maps are output through convolutional layers, and adjusted by the Scale module. During the training phase, the output is a set of feature maps; during inference, the feature maps are concatenated, and the final detection results are generated based on preset dynamic or fixed shapes.

The DAIH module significantly improves the model’s performance in object detection through innovative task decomposition and dynamic alignment techniques. Its reasonable architectural design and clear module functions enable the detection head to provide efficient and accurate detection results in complex detection tasks. Future research can further optimize the parameters and design of each module to enhance the detection head’s efficiency and accuracy, offering more possibilities for the development of object detection.

#### Design of context-sensitive modulation.

To further enhance the network’s representation capability, we have innovatively proposed a context-sensitive modulation (CSM) module. This module achieves deep enhancement of the input features through multi-level context aggregation and modulation.

**Fig 5:**
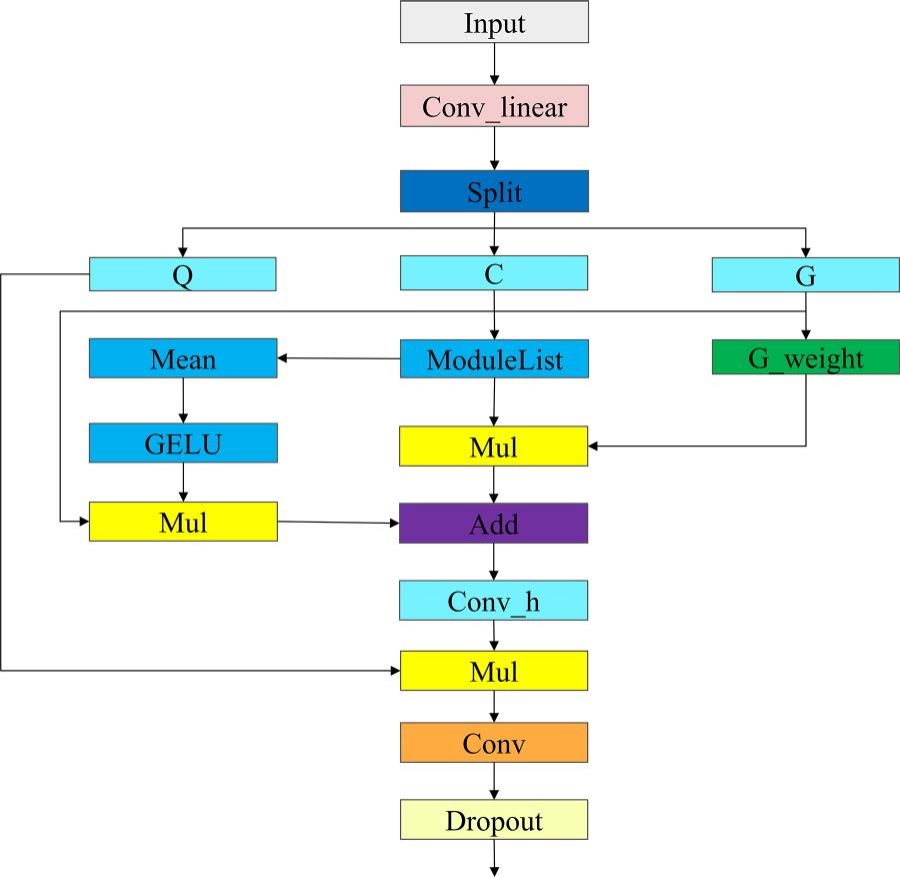
Algorithm schematic diagram of the CSM module.

The core of context-sensitive modulation lies in its unique architectural design, as illustrated in the algorithmic framework diagram shown in Fig 5. The input feature *x* is decomposed into a query Q, context C, and gating coefficients G through a preliminary linear projection layer, Conv_linear. This decomposition allows the model to process and modulate different types of information separately. In the multi-level focal convolution layer, we introduce several convolution layers with progressively larger kernels. Through this design, the contextual information at each level can be effectively aggregated and multiplied by the corresponding gating coefficients, achieving layer-by-layer accumulation. The focal convolution layers in the diagram demonstrate how context information is aggregated at each level. Specifically, to capture global context information, we obtain global context through average pooling and multiply it by the final gating coefficient before adding it to the final context representation. After context aggregation, normalization is performed to balance the importance of context from different levels, ensuring a more uniform overall context. Next, the query Q is modulated with the normalized context through element-wise multiplication, effectively integrating the multi-level context information into the query. To further improve the model’s performance, we introduce a posterior linear projection layer after modulation. This layer performs a linear transformation on the modulated output and increases the model’s robustness and generalization ability through a dropout layer [23]. If posterior layer normalization is enabled, layer normalization will be applied at this stage to further stabilize the output.

The context-sensitive modulation achieves deep enhancement of the input features by introducing multi-level context aggregation and modulation. This approach not only significantly enriches the feature representation but also enhances the overall expressive capability of the network. The CSM mechanism not only excels in visual tasks but also shows its potential and advantages in a wide range of applications. The architecture diagram illustrates the complete process from input to output, intuitively showing how the focal modulation mechanism progressively enhances the input features through various steps. This design is not only theoretically innovative but also demonstrates excellent performance and application prospects in practice.

#### Backbone network repvit.

The backbone network RepViT primarily employs transfer learning methods, leveraging the knowledge of pre-trained models to achieve rapid convergence and significantly enhance performance on new tasks [24]. In infrared target detection tasks, the introduction of RepViT significantly improved YOLOv8’s detection accuracy and real-time performance. Specifically, RepViT optimized the macro-architectural elements of the network, such as the initial layers, downsampling layers, and classifiers, achieving more efficient feature processing and target recognition. This is particularly important for infrared images in complex backgrounds and diverse target scenarios, enabling the system to excel in various challenging application environments.

By adopting RepViT as the backbone network, YOLOv8 demonstrated high accuracy and low inference latency in practical applications, particularly excelling on mobile devices and embedded systems. This innovation not only expands YOLOv8’s application prospects in infrared target detection but also provides new insights and directions for research in related fields.

#### Three loss functions are proposed.

In infrared target detection, to improve the YOLOv8 model’s detection accuracy and localization precision, three novel loss functions—focalerDIoU, focalerGIOU, and focalerShapeIoU—were proposed. FocalerGIOU integrates IoU with the smallest enclosing box area, enabling effective optimization even with non-overlapping boxes by providing a broader global measure. FocalerDIoU combines IoU with center point distance, enhancing box positioning accuracy. FocalerShapeIoU extends this by incorporating shape matching alongside IoU and center distance, optimizing the predicted box across overlap, position, and shape dimensions. These enhancements boost accuracy and robustness in infrared target detection. Developed from the Focaler-IoU concept [25], these functions innovatively blend bounding box adjustment strategies from GIoU [26], DIoU [27], and ShapeIoU [28]. The principle of focalerShapeIoU is outlined below:


$$IoU=\frac{Intersection\;Area}{Union\;Area}$$ (1)

$$Focaler\_IoU=(\frac{IoU-d}{u-d}).clamp(0,1)$$ (2)

$$distance=\frac{center\_distance}{cw2+ch2+\epsilon }$$ (3)

$$shape\_cost={\left(1-exp\left(-\frac{|w_{1}-w_{2}|}{max(w_{1},w_{2})}\right)\right)}^{4}+{\left(1-exp\left(-\frac{|h_{1}-h_{2}|}{max(h_{1},h_{2})}\right)\right)}^{4}$$ (4)

ShapeIoU=IoU−distance−0.5·shape_cost (5)

$$focalerShapeIoU=(\frac{ShapeIoU-d}{u-d})clamp(0,1)$$ (6)

Formula (1) calculates IoU to measure overlap between the predicted and target boxes. Formula (2) defines Focaler_IoU, where adjustment parameters *d* and *u* scale IoU, clamped between 0 and 1. Formulas (3) and (4) compute the distance and shape loss components of ShapeIoU, assessing box shape similarity via width and height differences, with exponential functions and higher powers enhancing sensitivity. Formula (5)yields the ShapeIoU loss, while formula (6)details the derivation of focalerShapeIoU. This loss function integrates shape differences for precise bounding box similarity assessment and leverages the Focaler-IoU strategy to focus on difficult samples, boosting training effectiveness.

The focalerDIoU loss function combines IoU with the center point distance, aiming to more effectively assess and optimize the localization of the detection box. The principle of DIoU is shown in formula (7), where ρ is the Euclidean distance between the center points of the predicted box and the target box, and c is the diagonal length of the minimum enclosing box surrounding the predicted and target boxes.


$$DIoU=IoU-\frac{\rho^{2}}{c^{2}}$$ (7)

$$focalerDIoU=(\frac{DIoU-d}{u-d})clamp(0,1)$$ (8)

The principle of focalerDIoU is shown in formula (8). Building on the original DIoU’s consideration of the minimum enclosing box for bounding boxes, focalerDIoU enhances the focus on difficult samples using the Focaler-IoU focal loss strategy, effectively improving the model’s detection performance by more accurately assessing the similarity of bounding boxes.


$$GIoU=IoU-\frac{A_{C}-A_{U}}{A_{C}}$$ (9)

$$focalerGIOU=(\frac{GIoU-d}{u-d})clamp(0,1)$$ (10)

The principle of the GIoU loss function is shown in Formula (10). Here, Ac is the area of the minimum enclosing box surrounding the predicted and target boxes, and Au is the area of the union of the predicted and target boxes. FocalerGIOU considers global information by incorporating the area of the minimum enclosing box, providing a more meaningful metric when the boxes do not completely overlap. The principle of focalerGIOU is shown in Formula (10).

## 5. Experimental results and analysis

### 5.1 Datasets and training configurations

**Fig 6:**
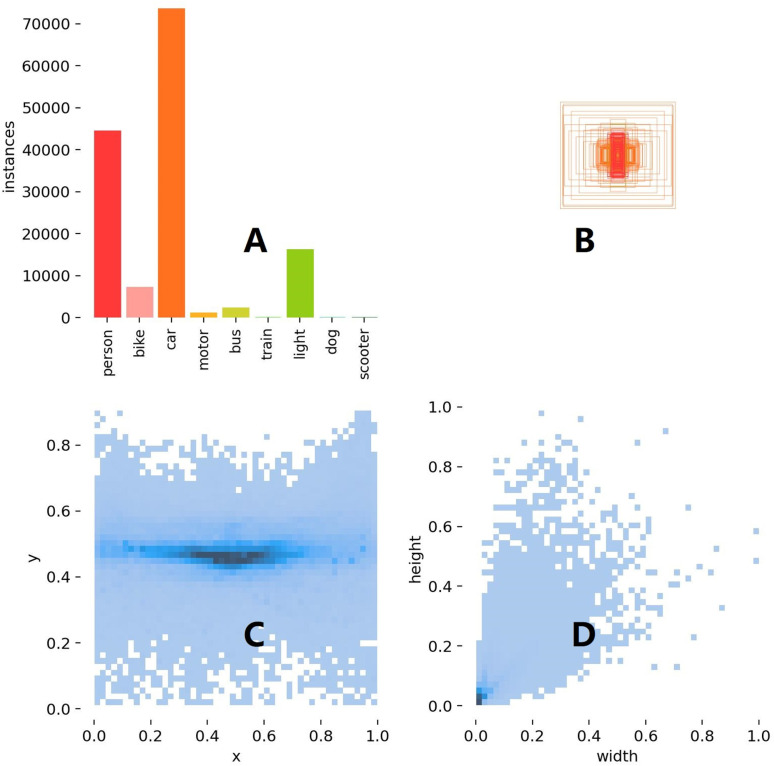
Number of categories and distribution of categories in the dataset.

The dataset used in this experiment is the FLIR_ADAS_v2 version, released in 2022. It contains 9,233 visible spectrum images, 9,711 thermal spectrum images, and 7,498 matched thermal-visible video frames. The FLIR ADAS v2 dataset is an enhanced and expanded thermal imaging dataset, specifically designed for the development and testing of Advanced Driver Assistance Systems (ADAS) and autonomous driving technologies. The dataset includes over 26,000 images captured under various conditions. In this experiment, the focus is on the training, testing, and validation sets of thermal images. The training set (images_thermal_train) contains 8,862 images, the validation set (images_thermal_val) contains 1,366 images, and the testing set (images_thermal_test) contains 3,674 images. The experiment primarily involves nine categories selected from the training set, Fig 6 shows the data distribution in the target detection task. Fig 6-A is a bar chart showing that the category ‘person’ has the highest number of instances, close to 70,000, followed by ‘bike’ with about 50,000, and other categories like Fig 6-B is an illustration of the distribution of the prediction boxes, and the size and position of the prediction boxes; Fig 6-C is a two-dimensional scatter plot showing the dense distribution of target width and height, with dark banded areas in the middle; Fig 6-D is similar to Fig 6-C, but with a more dispersed distribution, with points concentrated in the lower left. is more dispersed, with points concentrated in the lower left corner and in the middle, indicating the diversity of target sizes under different conditions.

For the classification results of small, medium, and large objects in the dataset, this paper defines a small object as one whose bounding box area is less than 32*32 pixels, a medium object as one whose pixel range is more than 32*32 pixels but less than 96*96 pixels, and a large object as one whose area exceeds 96*96 pixels. Table 1 uses the statistical results from the validation set in the dataset. From Table 1, it can be seen that small objects account for a larger proportion in each category of the dataset, with the total proportion of small objects being 87.9%. The proportion in each category also exceeds 70%, indicating that the dataset is predominantly composed of small objects.

**Table 1:** Statistics of Large, Medium, and Small Targets.

Category	Small Object Count	Small Object Percentage	Medium Object Count	Medium Object Percentage	Large Object Count	Large Object Percentage	Total Annotations
person	4090	94.92%	208	4.83%	11	0.26%	4309
bike	144	84.71%	26	15.29%	0	0.0%	170
car	5674	79.55%	1358	19.04%	101	1.42%	7133
light	1999	99.7%	6	0.3%	0	0.0%	2005
bus	128	71.51%	43	24.02%	8	4.47%	179
motor	41	74.55%	13	23.64%	1	1.82%	55
total	12076	87.19%	1654	11.94%	121	0.87%	13651

For the preprocessing part of the dataset, since the dataset FLIR uses a data type that is not in YOLO format, the dataset was analysed by converting the FLIR json annotation file provided in the dataset index.json to the YOLO tags for each image by employing a script file. And create a.txt file containing all the image names extracted from the JSON file and append the specified directory to its name. The dataset previews the images with class name tags during the conversion process so that the user can visually check the validity of the data. For the output of the dataset conversion process, a folder named labels is created where the script resides within the FLIR dataset folder. This will contain label txt files named with the same image names provided by FLIR, each.txt file will contain bounding boxes and classes in YOLO format, a.txt file called all_image_names.txt, which will contain all the images in the dataset file with the input directory appended to its name, and by preprocessing the dataset, the FLIR dataset into YOLO format, for is dataset preprocessing, as the categories of detection can be set in advance in the script file, in this paper, we have selected nine to be detected by the categories in the dataset and the number of each category, and the dataset is preprocessed by these operations.

Several parameters need to be conFigd during the model training process, as shown in Table 2. The SGD optimizer was used, with a Momentum value of 0.937, a learning rate of 0.01, a Batch Size of 16, 100 Epochs, and a weight_decay value of 0.0005. The server configuration includes 100 GiB of memory, 30 GiB of video memory, and one NVIDIA A10 GPU. The environment configuration includes modelscope 1.13.1, pytorch 2.1.2, tensorflow 2.14.0, and ubuntu 22.04.

**Table 2:** Experimental environment and parameter configuration strategy.

Training Parameters	Value
operating system	**Ubuntu22.04**
GPU	**NVIDIA A10**
framework	**Pytorch 1.13.1**
input size	**640**
batch size	**16**
epoch	**100**
learning rate	**0.01**
momentum	**0.937**
optimize	**SGD**

SGD offers stability and strong generalization ability in object detection tasks, efficient convergence when combined with momentum, lower computational overhead, compatibility with the YOLO series, and adaptability to the noise and complex backgrounds encountered in infrared small target detection tasks. Although ADAM is favored in some tasks for its rapid convergence, in the context of MDCFVit-YOLO, SGD is more suitable for maintaining a balance between accuracy and real-time performance while effectively supporting the optimization of innovative modules (MPA, CSM, and DAIH) and loss functions.

### 5.2 Evaluation of indicators

In this paper, Precision (P), Recall (R) and mean Average Precision (mAP) are used as metrics to evaluate the performance of the model. The evaluation index formula is as follows:


$$p=\frac{TP}{TP+FP}$$ (11)

$$R=\frac{TP}{TP+FN}$$ (121)

$$AP=\int_{0}^{1}PRdr$$ (13)

$$mAP=\frac{1}{n}\sum_{i=0}^{n}AP_{i}$$ (14)

In Eqs. (11)–(14), TP denotes the number of positive samples correctly recognized as positive classes, FP denotes the number of negative samples incorrectly recognized as positive classes, FN denotes the number of positive samples incorrectly recognized as negative classes, and n denotes the total number of samples and the number of classes in the dataset. n represents the total number of samples and the number of classes in the dataset. Flops can reflect the complexity of the model to some extent, and FPS indicates the number of images detected per second, and these two metrics can be used as important parameters for evaluating the model.

### 5.3 Ablation experiments

To verify the effect of the MDCFVit-YOLO model, the ablation experiments are carried out for each improved scheme under the same strategy, the results are shown in Table 3. The FPS is added as a reference index for the model. In order to facilitate the use of the reader to understand the meaning of Table 3, for example, for model1, is in the YOLOv8 based on the improvement of the obtained, for the improvement of which, the reader can see in Table 3, tick is in the YOLOv8 based on the improvement of the part, you can see that model1 is the repvit model is used in the YOLOv8, and the model1 is used in YOLOv8. The other parts of the table are also the same as model1 is the same form of expression.

**Table 3:** Experimental results of the ablation experiment.

Model	Repvit	MPA	DAIH	CFM	Loss	Precision	Recall	mAP@0.5	Parameters	FPS
YOLOv8n						**0.735**	**0.529**	**0.606**	**6.2**	**249.5**
Models1	**□**					**0.734**	**0.562**	**0.638**	**13.4**	**116.7**
Models2		**□**				**0.75**	**0.54**	**0.622**	**11.8**	**167**
Models3			**□**			**0.747**	**0.551**	**0.633**	**11.9**	**129.8**
Models4				**□**		**0.744**	**0.542**	**0.618**	**6.2**	**239.3**
Models5					**□**	**0.728**	**0.537**	**0.618**	**6**	**251.2**
MDCFVit-YOLO	**□**	**□**	**□**	**□**	**□**	**0.78**	**0.586**	**0.67**	**20.9**	**65.2**

Models1 adopts Repvit to replace the YOLOv8 backbone model by modifying the YOLOv8 backbone. Compared with the baseline model, the mAP and recall rates are improved by 3.2% and 3.3%, respectively. Repvit uses the strategy and method of employing a complex network structure to enhance the learning ability during the training process, and simplifying the structure to improve the computational efficiency in the inference stage, therefore, misjudgment is reduced and the detection accuracy is improved. The multiplexed interactive attention mechanism is used for Models2, the detection accuracy is increased by 1.6% compared with Models1 while the number of parameters is slightly decreased. Compared with the YOLOv8 model, the mAP is increased by 1.6%. Models3 uses a dynamic automated detection head to improve the detection head part of YOLOv8, the mAP is 63.3%, and the mAP and precision are improved by 2.7% and 1.2% compared to YOLOv8, respectively. Models4 replaces the traditional SPPF with the CFM module and optimizes its structure, resulting in the mAP, precision, and recall rate being improved by 1.2%, 1%, and 1.3% respectively. A new focalerShapeIoU loss function is used for Models5, the mAP and recall are improved by 1.2% and 1% through optimizing the border of the model, respectively. Although the model parameters decreased compared with Yolov8, the fastest processing speed is 251.2 FPS. The detection precision is 75.1% for the MDCFVit-YOLO network, which is 1.6% higher than the baseline model, the recall is 58.8% and the mAP is 66.7% which is 5.9% and 6.1% is higher than the baseline model, respectively. Due to the significant performance improvement of the model, the increase of 20.9M for the computation of the model and the decrease of 65.2 FPS for the computation speed are also acceptable.

MDCFVit-YOLO performs best in detecting people and car categories, with mAP50 reaching 77.3% and 85.1% respectively. The mAP50-95 for the car category even reaches 60.7%, demonstrating high detection accuracy and stability. However, the performance is poorer in detecting bicycles and traffic lights, with a significant drop in accuracy, where mAP is only 48.1% and 57.1% respectively. Detailed detection data for each category can be seen in Table 4. Overall, the model performs well in common traffic scenarios, but there is room for improvement in the recognition of details and small objects.

**Table 4:** Detection Performance of the MDCFVit-YOLO Model on Various Categories.

Class	Images	Instances	Box (P)	R	mAP50	mAP50–95
**all**	**1144**	**13843**	**0.78**	**0.586**	**0.67**	**0.405**
**person**	**1144**	**4309**	**0.812**	**0.675**	**0.773**	**0.442**
**bike**	**1144**	**170**	**0.57**	**0.512**	**0.481**	**0.294**
**car**	**1144**	**7128**	**0.832**	**0.783**	**0.851**	**0.607**
**motor**	**1144**	**55**	**0.89**	**0.586**	**0.683**	**0.359**
**bus**	**1144**	**179**	**0.801**	**0.497**	**0.66**	**0.473**
**light**	**1144**	**2002**	**0.775**	**0.463**	**0.571**	**0.252**

### 5.4 Loss function comparison experiment

The focalerDIoU, focalerGIOU, focalerShapeIoU, GIoU, and DIoU are used for comparison experiments to study the most suitable loss function as infrared target detection, respectively. The metrics of the loss functions are shown in Table 5.

**Table 5:** Comparative experimental data of loss functions.

IoU_Loss	Precision	Recall	mAP@0.5
focalerDIoU	**0.738**	**0.55**	**0.617**
focalerGIOU	**0.739**	**0.523**	**0.616**
GIoU	**0.708**	**0.556**	**0.617**
DIoU	**0.727**	**0.552**	**0.606**
focalerShapeIoU	**0.789**	**0.534**	**0.626**

**Fig 7:**
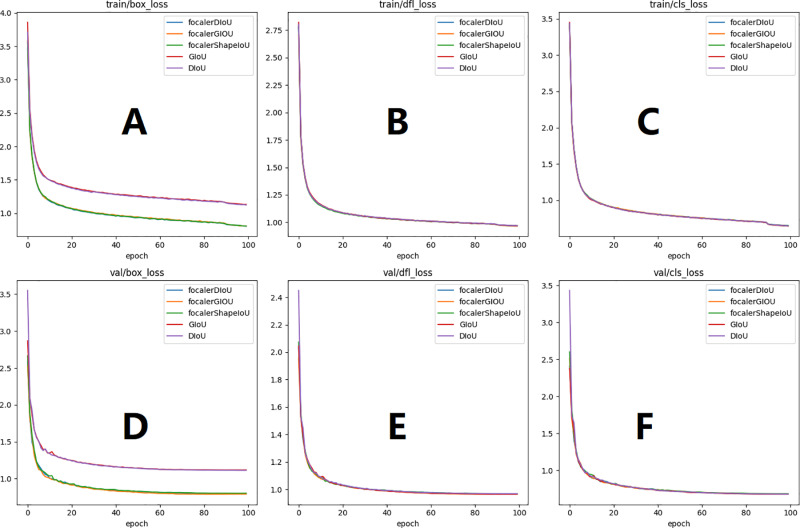
Loss function curve graphs. Figs 7(A), 7(B), and 7(C) show the training set losses, Figs 7(D), 7(E), and 7(F) show the validation set loss curves. Figs 7(A)、 7-(D) represent localization loss, 7-(B)、7-(E) represent confidence loss, 7-(C)、7-(F) represent classification loss.

**Fig 8:**
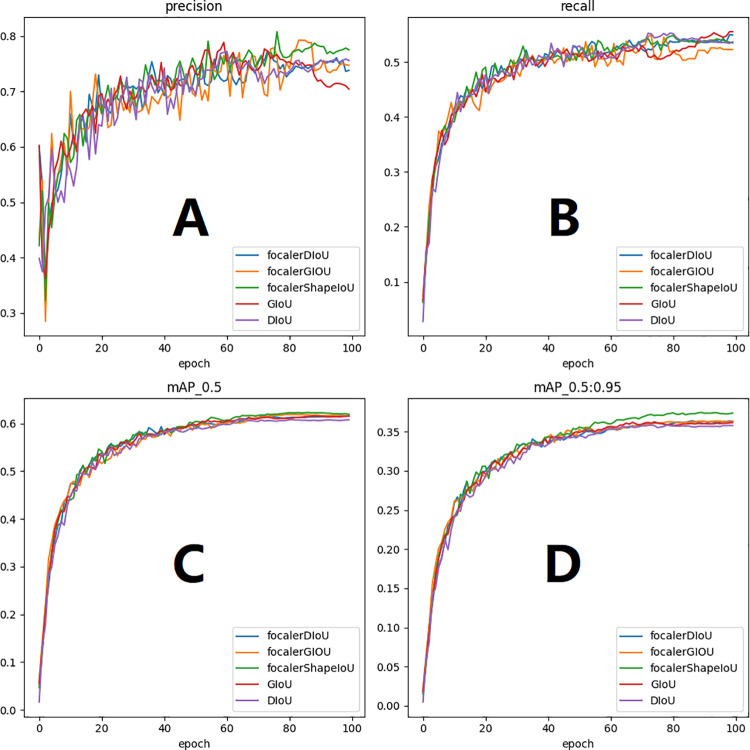
Evaluation metrics graphs for different loss functions. Fig 8(A) is precision, Fig 8(B) is recall, Fig 8(C) is mAP50, Fig 8(D) is mAP95.

As can be seen from Table 5 the mAP value of GIoU is 61.7% and almost the same as that of focalerGIOU, and focalerDIoU, the mAP value of DIOU is the smallest at 60.6%, and focalerShapeIoU has the best mAP value as high as 62.6%. It is found that the detection accuracy of focalerShapeIoU is optimal as high as 78.9%, which is improved by 5.1%, 5%, 8.1%, and 6.2% compared to focalerDIoU, focalerGIoU, GIoU, and DIoU, respectively. Therefore, the performance of focalerShapeIoU is optimal. To clearly show the effects of different loss functions on the infrared target detection, the changes of different loss functions on the loss curves during training and the effects on the evaluation index of the detected objects in the detection of Infrared targets are shown in Fig. 7 and Fig. 8.

From Fig 7(B), Fig 7(C), Fig 7(E), Fig 7(F), it can be seen that the trend of the five loss functions of classification loss and confidence loss is the same, but from Fig 7(A), Fig 7(D) it can be seen that the loss of the loss change curve of the GIoU and the DIoU is relatively large. Therefore, the GIoU and DIoU losses are rounded off and not considered as the loss function of the improved YOLOv8.

As shown in Fig 8 all curves exhibit a rapid increase and eventually stabilize. The green line represents the focalerShapeIoU metric indicators, which is almost on top of the chart in the Precision, Recall, and mAP.Therefore, the focalerShapeIoU is used as the loss function for the improved YOLOv8 as a result of comprehensive consideration. From the above results, it is evident that focalerShapeIoU as the new loss in infrared target detection is beyond doubt. However, the proposed focalerGIOU and focalerDIoU loss functions also perform excellently, although slightly lower than focalerShapeIoU in this task. These two functions might outperform focalerShapeIoU in other detection tasks or computer vision experiments.

### 5.5 Comparative experiments and their visualization

To further validate the effectiveness and superiority of the proposed algorithm, we tested several state-of-the-art target detection algorithms on the FLIR dataset, with experimental data and detection results visualized for analysis. The performance metrics of each model are presented in Table 6. These models were evaluated based on key indicators, including precision, recall, mAP, and the number of parameters.

**Table 6:** Model comparison test results.

Model	Precision	Recall	mAP@0.5	Parameters(M)	FPS
YOLOv3	**0.69**	**0.443**	**0.493**	**23.2**	**153.7**
YOLOv5	**0.751**	**0.521**	**0.61**	**5**	**186.1**
YOLOv6	**0.7525**	**0.523**	**0.588**	**8.3**	**161.3**
YOLOv7	**0.741**	**0.522**	**0.612**	**12.3**	**170.9**
YOLO8	**0.735**	**0.529**	**0.606**	**6.2**	**210.4**
YOLOv10	**0.698**	**0.509**	**0.58**	**5.5**	**229.2**
MDCFVit-YOLO	**0.78**	**0.586**	**0.67**	**20.9**	**65.2**

Comparative experiments on infrared detection were conducted using YOLOv3, YOLOv5, YOLOv6, YOLOv7, YOLOv8, YOLOv10, and MDCFVit-YOLO. Table 6 shows that MDCFVit-YOLO achieves a precision of 0.78, surpassing other models such as YOLOv5 (0.751), YOLOv6 (0.7525), YOLOv7 (0.741), and YOLOv8 (0.735), with improvements ranging from 3.86% to 6.12%. This indicates superior accuracy in identifying infrared targets. In terms of recall, MDCFVit-YOLO reaches 0.586, outperforming YOLOv5 (0.521), YOLOv6 (0.523), YOLOv7 (0.522), and YOLOv8 (0.529) by 6.25% to 12.47%, effectively reducing missed detections. The mAP@0.5 of MDCFVit-YOLO is 0.67, a significant improvement over YOLOv5 (0.61), YOLOv6 (0.588), YOLOv7 (0.612), and YOLOv8 (0.606), with a 9.48% gain compared to YOLOv7, highlighting enhanced overall detection performance. Although MDCFVit-YOLO has 20.9M parameters—higher than YOLOv5 (5M), YOLOv6 (8.3M), YOLOv7 (12.3M), and YOLOv8 (6.2M)—the substantial performance gains justify the increased complexity, striking a balance between model size and effectiveness. These results underscore MDCFVit-YOLO’s superiority in infrared target detection, providing a robust foundation for further research and applications.

**Fig 9:**
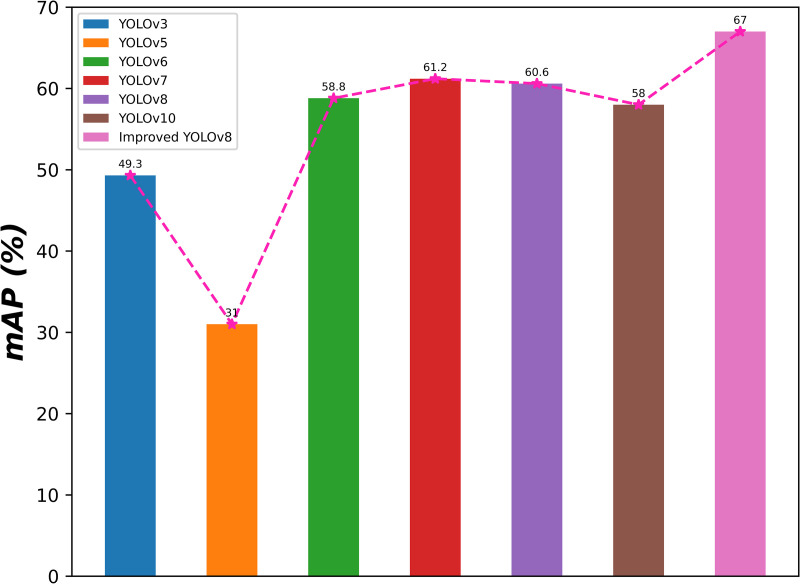
YOLO series algorithms in the detection of infrared target city mAP value results of the map.

**Fig 10:**
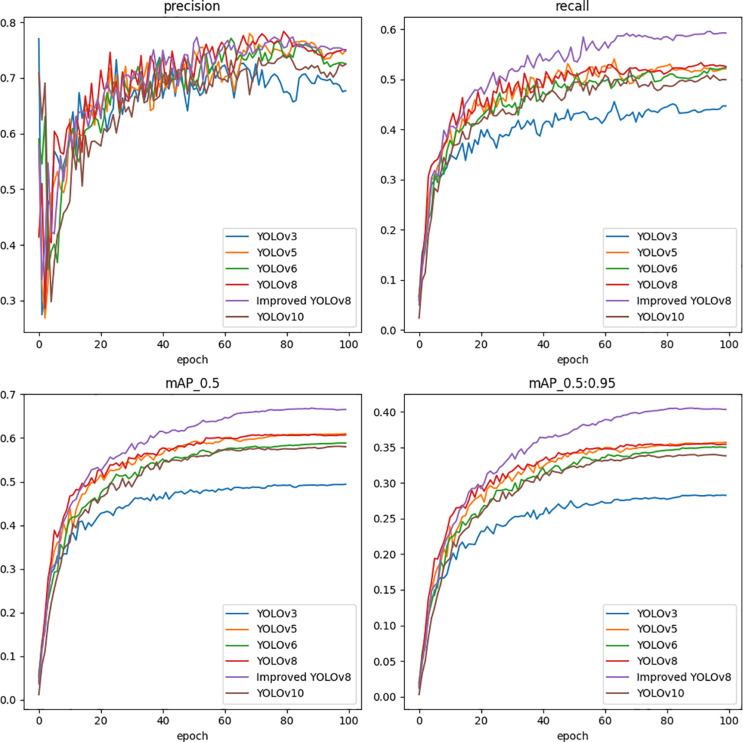
Results of YOLO series models in 100epoch in terms of variation in precision, recall, mAP50, and mAP95.

To clearly show the detection effect, the mAP of all contrast models is visualized as shown in Fig 8. It is clear from Fig 9 that the MDCFVIT-YOLO model performs best in infrared target detection. To clearly show the training results of precision, recall, mAP50, and mAP95 of each comparative model during each round of training, visualization results of the 100 rounds of experimental data of the YOLO series of models in the detection of infrared targets are shown in Fig. 10. As can be seen from Fig 10, all evaluation indicators of the proposed model far exceed those of other YOLO series detection algorithms, especially in both accuracy and recall rate.

**Fig 11:**
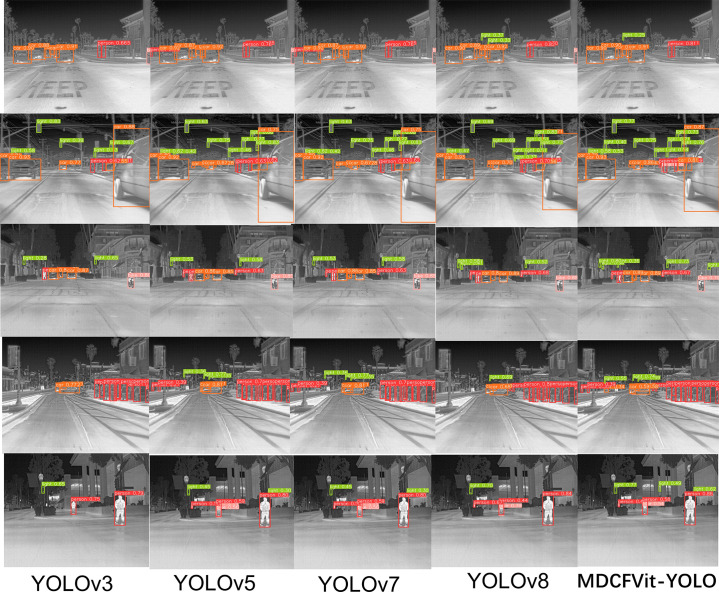
Contrast the visualization results of the experiment.

To highlight the detection effect of the MDCFVIT-YOLO model, the visual contrast experiment of the detection effect of the Yolo series detection algorithm is carried out, results are shown in Fig 11.

Infrared object detection performance varies significantly across models. Comparing YOLOv3, YOLOv5, YOLOv7, YOLOv8, and the proposed MDCFVit-YOLO model, as shown in Fig 11, reveals distinct differences. YOLOv3 struggles with frequent missed and false detections, particularly in complex backgrounds, resulting in unstable and poor performance. YOLOv5 and YOLOv7 improve accuracy and reduce errors compared to YOLOv3, but they still fall short in target boundary precision and small target detection. YOLOv8 enhances accuracy and speed, yet it cannot fully eliminate missed detections or boundary issues in certain infrared scenarios. In contrast, the MDCFVit-YOLO model excels in these test cases, effectively capturing multi-scale target features and maintaining high accuracy in complex backgrounds. It minimizes missed and false detections, offering superior boundary precision and small target detection capabilities. Overall, MDCFVit-YOLO outperforms the others, delivering more accurate, stable, and reliable results, marking a significant advancement in infrared target detection.

**Fig 12:**
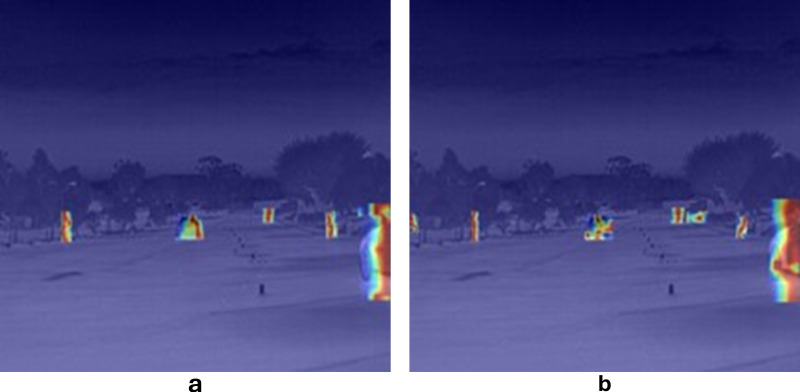
Model thermal diagrams (a) for the YOLOv8 model (b) for the MDCFVit-YOLO model.

Fig 12 shows the feature visualization of YOLOv8 and the proposed model detection results in infrared scenes. The experimental results clearly show that the network of feature extraction effectiveness on images, of which, the MDCFVit-YOLO model performs better in target recognition and has a higher recognition rate.

## 6. Conclusion

This paper proposes the MDCFVit-YOLO model by introducing new backbone networks and detection heads, along with a self-developed model, enhancing the accuracy and real-time performance of infrared vehicle and pedestrian detection. Experimental results demonstrate that the MDCFVit-YOLO model achieves a precision of 78%, a recall of 58.6%, and an mAP of 67%, significantly outperforming other YOLO series models. For small target detection in complex nighttime environments, the model excels in addressing challenges such as low visibility and high interference. Under conditions of varying illumination, multi-target detection, and small target scenarios, it markedly improves small target detection accuracy. This study offers new ideas and methods for advancing infrared vehicle and pedestrian detection. Future research can focus on optimizing the model structure, enhancing detection speed and efficiency, and extending its application to more complex infrared target detection scenarios.
